# Incidentally Found Prostate Cancer and Influence on Overall Survival after Radical Cystoprostatectomy

**DOI:** 10.1155/2012/690210

**Published:** 2012-06-03

**Authors:** Algimantas Sruogis, Albertas Ulys, Giedre Smailyte, Zygimantas Kardelis, Arunas Kulboka, Giedre Anglickienė, Nerimantas Samalavicius, Marius Anglickis

**Affiliations:** ^1^Department of Urology, Institute of Oncology, Vilnius University, Santariški*ų* 1, 08660 Vilnius, Lithuania; ^2^Cancer Control and Prevention Center, Institute of Oncology, Vilnius University, Santariški*ų* 1, 08660 Vilnius, Lithuania; ^3^Clinic of Surgery, Institute of Oncology, Vilnius University, Santariški*ų* 1, 08660 Vilnius, Lithuania

## Abstract

*Objectives*. To determine incidentally found prostate cancer frequency and impact on overall survival after RCP. *Patients and Methods*. The records of 81 men who underwent cystoprostatectomy from January 2000 to December 2009 were reviewed. The vital status of the study group was assessed as on September 1, 2009, by passive followup, using data from the population registry. *Results*. The 81 men underwent RCP. The incidental prostate cancer was found in the specimens of 27 (33.3%) patients. 13 (48.1%) of 27 prostate cancer cases were clinically significant. For 3 patients (11.1%) an extraprostatic extension was found. For 2 patients (7.4%)—positive margins, for 1 patient (3.7%)—Gleason sum 8, and for the rest 7 patients bigger than 0.5 cm^3^ volume tumor, and Gleason sum 7 was found. The mean follow-up time was 39.2 ± 33.8 months (varies from 0.8 to 131.2 months). The patients with bladder cancer and incidentally found prostate cancer lived shorter (28.1 ± 27.5 and 45.5 ± 35.40 months). Higher overall survival (*P* = 0.03) was found in the patient group with bladder cancer without incidentally diagnosed prostate cancer. *Conclusion*. There are indications that in this small study prostate cancer has influenced on patients' survival with bladder cancer after radical cystoprostatectomy.

## 1. Introduction

Bladder cancer is the second most common cancer of urinary tract after prostate cancer and the fourth most common malignancy in men [1]. Although the disease may occur in young persons, about 78% of all cancers are diagnosed in persons of age 55 years and older [2]. 70% of all patients with bladder cancer have superficial cancer that does not reach the muscular layer, and most of these patients have a fairly good prognosis. Most of the patients with superficial bladder cancer have pTa bladder cancer stage. 20% of all patients with bladder cancer have pT1 bladder cancer stage and just 10% is carcinoma in situ [3]. Patients with carcinoma in situ have the biggest risk of cancer progression into the muscular layer and also are in the biggest risk group of death. Radical cystoprostatectomy (RCP) is the standard and effective treatment method for the patients with invasive or superficial recurrent bladder cancer who are in a high progression risk group. Patients' survival after RCP depends on the primary tumor grade and stage. 5-year survival after RCP varies from 33 to 73%, and no other medical attempts during the last 10 years had no influence for patients' survival [4]. The standart technique of RCP in men consists of removing a bladder together with the removal of a prostate, seminal vesicles, a part of the vasa deferentia and distal ureter, including regional lymphadenectomy. In common cases, RCP is related with a sexual and urinary disfunction. In order to preserve patients' erectile function, urination and the quality of life associated with RCP, the new sparing techniques of prostate have been described, including procedures that spare the apex, the prostatic capsule and seminal vesicle, and even the total prostate with neurovascular bundle [5–7]. Preserved autonomic and sensory pelvic nerves can determine better urination and potency results. On the other hand, all these modifications of RCP are associated with non radical cancer elimination in those cases when prostate cancer or local urotel carcinoma invasion to prostate or prostatic uretra part was not diagnosed before the operation [8, 9]. After RCP, incidental prostate cancer is diagnosed from 23 to 54% of patients and has not clear clinical influence [10, 11]. Incidentally found prostate cancer is classified into two groups: clinical significant and clinical insignificant prostate cancer. This classification does not show the exact malignancy of prostate cancer because it is caused by many other factors [12, 13]. Prostate cancer is clinical significant when there is positive tumor margins, extraprostatic extension, Gleason score is more than 6 or tumor volume is 0.5 cm^3^ or bigger. Perineural invasion is one of clinical significant prostate cancer signs which show a biological malignancy and recurrence risk [14, 15]. There are not many studies where the influence of incidentally found prostate cancer for patients' overall survival was examined. In those studies, where the influence of incidentally found prostate cancer for overall survival was examined, the majority of all clinical significant prostate cancer cases could not be stratified as clinically significant because those cases did not satisfy with the latter-day clinically significant prostate cancer conception. With this retrospective analysis, we wanted to evaluate incidentally found prostate cancer frequency after RCP, histopathological characteristics of it, and incidental prostate cancer impact on patients' overall survival.

## 2. Patients and Methods

The records of 81 men who underwent RCP for transitional cell carcinoma of a bladder at our institution from January 2000 to December 2009 were reviewed. The incidental prostate cancer was found in the specimens of 27 (33.3%) patients (with a mean age at surgery of 61.3 ± 8.0), and their pathological and clinical records were analyzed. 7 patients with a known history of prostate cancer were excluded. A routine pathological examination was used for all RCP specimens, by sectioning and totally submitting the bladder and prostate tissue. Soon after the operation, the prostate was severed from the bladder and then prostates were weighed, inked, and fixed in 10% formalin. After fixation the prostate specimens were sectioned at 3 mm intervals perpendicular to the apical-basal axis of the gland. The seminal vesicles were sectioned parallel to their junction with the prostate. The volume of cancer was determined by the grid-counting method [16, 17]. The sum of the area of each slice was multiplied by the average slice thickness, and the sum of these volumes was multiplied by 1.25 to account for formalin shrinkage. All cancers were graded according to the Gleason system. The 1997 TNM (tumor, nodes, and metastasis) system was used for pathologic staging. Surgical margins were considered positive when cancer cells were in contact with the inked margin. Extraprostatic extension was defined as cancer extending into extraprostatic tissue. The numeric variables were presented as mean ± SD. The vital status of the study group was assessed as on September 1, 2009, by passive followup, using data from the population registry. It was found that 20 (33.9%) of the patients had died. Survival was estimated by the Kaplan-Meier method, and differences were assessed with the log-rank statistic. *P* < 0.05 indicated a significant statistical difference. For between groups, comparison of categorical variables was used chi-square test. All statistical analyses were performed using Stata Statistical Software version 11 (StataCorp. 2009. Stata Statistical Software: Release 11. College Station, TX, USA).

## 3. Results

The mean age of patients with prostate cancer was 60.6 years; without prostate cancer 62.8 years. Statistically significant difference between patients age in those two groups was not found. Incidentally found prostate cancer rate in investigated group was 33.3% (27 patients).  Table 1 shows the histopathological characteristics of prostate cancer. For 14 patients (51.8%), tumor was found in both prostate's lobes, and for the rest 10 (48.2%) patients prostate cancer was diagnosed just in one lobe. 13 (48.1%) of 27 prostate cancer cases were clinically significant. For 3 patients (11.1%) in clinical significant cancer group an extraprostatic extension was found during the histopathological examination. For 2 patients (7.4%)—positive margins, for 1 patient (3.7%)—Gleason sum 8, and for the rest 7 patients the bigger than 0,5 cm^3^ volume cancer with Gleason sum 7 was found during the final histopathological examination.

**Figure 1:**
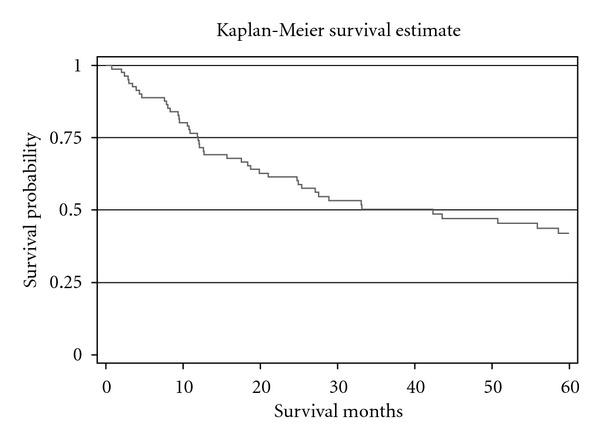


**Figure 2:**
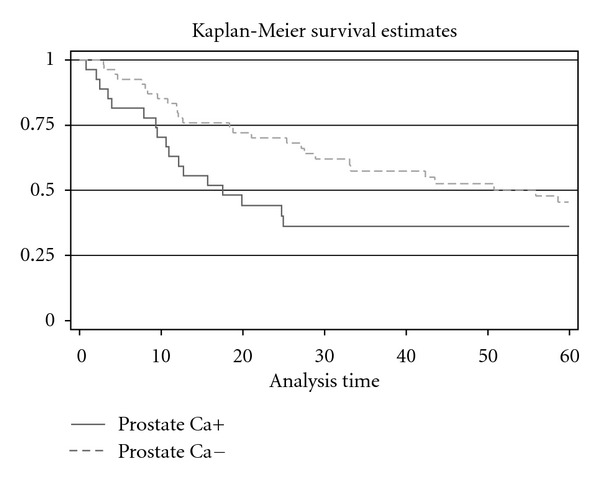


19 patients (70.3%) had prostate cancer with Gleason sum 6. Gleason sum 7 was diagnosed for 2 patients with pT3a bladder cancer and for 2 patients with pT2a bladder cancer. Clinically significant cancer was associated with higher Gleason sum, risk of extraprostatic extension and positive margins. For 3 cases from all 27 patients with prostate cancer were histopathologically diagnosed metastases from bladder cancer to lymphatic nodes.  Table 2 shows histopathological characteristic of bladder cancer. 3 patients (11.1%) with incidentally found prostate cancer had a superficial bladder cancer. For 2 patients (7.4%) from 27 patients group with prostate cancer, a pT2a bladder cancer stage was diagnosed. For 4 patients in this group (14.8%) pT2b, for 8 (29.6%)—pT3a, for 1 (3.7%)—pT3b, for 8 (29.6%)—pT4a, and for 1 (3.7%)—pT4b, bladder cancer stages were diagnosed. For 9 patients (16.6%) with bladder cancer and without prostate cancer, a superficial bladder cancer was diagnosed. For 10 patients in this group (18.5%)—pT2a, for 2 (3.7%)—pT2b, for 12 (22.2%)—pT3a, for 5 (9.2%)—pT3b, for 8 (14.8%)—pT4a, and for 8 (14.8%)—pTis, bladder cancer stages were diagnosed. Statistically significant difference between bladder cancer stage, cancer malignancy and positive lymphatic odes was not found between two groups. The mean follow-up time was 39.2 ± 33.8 months (varies from 0.8 to 131.2 months) (Figure 1). Patients with bladder cancer and incidentally found prostate cancer lived shorter than patients without prostate cancer (28.1 ± 27.5 and 45.5 ± 35.40 months). Higher overall survival (*P* = 0.03) was found in the patient's group with bladder cancer without incidentally diagnosed prostate cancer (Figure 2).

## 4. Discussion

Incidentally found prostate cancer is diagnosed during the pathological test after surgical intervention (radical cystoprostatectomy, etc.) for the patients without any symptoms or for those patients for whom this disease was not suspected during the physical examination (DRT) or the estimating laboratorial (PSA) and instrumental (prostate biopsy) tests. More than a half of all incidentally found prostate cancers and more than 90% of all prostate cancer influenced deaths occur for the patients who are 65 years old or older [10]. It is thought, that these facts are related with a worse Gleason's differentiation and clinical or pathological prostate cancer stage. Prostate cancer treatment with radical prostatectomy has limited use for older patients because of a higher risk of cancer metastasis till a disease is diagnosed [11]. The possible relation between prostate and bladder cancers is stated in many different studies [18–21]. Prostate cancer is diagnosed nine times more for men with bladder cancer. If not to count the mistakes of cases inclusion, prostate cancer is nineteen times more often between patients with bladder cancer than by age, sex, or race in adequate population [20]. Relation between these two cancers can be explained by genetical factors. It is known that the birth of both cancers is related with pathology of P53 and Rb genes.

The rate of incidentally found cancer is influenced by studied population. Lee et al. [22] shows that just for 4% of all 248 studied patients incidentally found prostate cancer was found in Taiwan. A bit more cases of incidentally found cancer were found in the Kurahashi et al. [23] study. Incidentally found cancer after RCP was diagnosed for 33.3% of all examined patients in our study. It is similar to many other studies which were made in Western Europe countries. In these studies, rate of incidentally found prostate cancer was from 23% to 51% [10, 11]. The most important criterion for incidentally found prostate cancer is clinically significant prostate cancer. There is no definition of clinically significant prostate cancer, but it is thought that it is related with a higher risk of local relapse or metastasis. The rate of clinically significant prostate cancer is from 14% to 53% [24, 25]. Such a wide variety of clinically significant cancer is influenced by studied population, the protocol of histopathological test and by the definition of clinically significant prostate cancer. The rate of clinically significant cancer in our study reaches 48.1%. For 3 (11.1%) patients extraprostatic extension, for 2 (7.4%)—positive margins, for 1 (3.7%)—Gleason sum 8 tumor, and for the rest 7—bigger than 0.5 cm^3^ volume tumor were diagnosed during histopathological tests. More and more often prostate sparing RCP, which improve sexual and urination function, are performed [20]. There are few authors, who recommend to perform prostate capsule or top sparing ECP for improving sexual and urination functions [5–7]. These operations can be related with neither the radical elimination of tumor nor a higher risk of positive margins and worse oncological results. By the way, that risk is higher than tumor is on the top of prostate. For 37.0% of all patients in our study, prostate cancer was found on the top of prostate. There are no many studies where influence for patients' survival of incidentally found prostate cancer after RCP was examined. Abdelhady et al. [14] found that the combination of prostate and bladder cancers has no influence on patients' survival prognosis and is related with one or another cancer stage. Pritchett et al. [26] also found that the patients with bladder cancer and incidentally found prostate cancer are not in a higher risk of death than patients who have only bladder cancer. Kouriefs et al. [27] in very small undertake study found that incidentally found prostate cancer is really frequent finding and has no influence for overall survival after radical elimination of cancer. Similar results were found in other studies [28, 29]. The problem is that clinically significant prostate cancer definition in these studies is contravening with nowadays conception, or mean time of patients observation was very short. There was no statistically significant difference between patients age, pathological stage, or number of positive lymphatic nodes in our study. Our study showed what overall survival for the patients with incidentally found prostate cancer was 28.1 ± 27.5 months versus  45.5 ± 35  (*p* − 0.03) for the patients without prostate cancer, respectively. Our study shows that incidentally found prostate cancer has an influence on overall survival, that is why it is important to pay more attention to this pathology. For all the patients for whom is planned to perform modified radical prostatectomy, prostate biopsy is recommended even if PSA concentration is normal. Prostate biopsy will allow choosing a more suitable treatment for bladder cancer, will reduce the risk of positive margins and the progression of prostate cancer, and will improve oncological outcomes. It is necessary to have more wide amount studies, which could confirm our findings.

## 5. Conclusion

Incidentally found prostate cancer is often found after the radical cystoprostatectomy. There are indications that in this small study prostate cancer has influence on patients' survival with bladder cancer after radical cystoprostatectomy. 

##  Conflict of Interests

The authors state no conflict of interests.

## Figures and Tables

**Table 1:** The histological characteristics of the 27 prostate cancers identified in the 81 RCP specimens.

	Number of patients	Significant tumour	Tumour at apex	Positive SM
	(% of total)	(% of total)	(% of total)	(% of total)
T stage (TNM)				
pT1a	1 (3.7)	0 (0)	0 (0)	0 (0)
pT2a	7 (25.9)	0 (0)	0 (0)	0 (0)
pT2b	2 (7.4)	1 (3.7)	0 (0)	0 (0)
pT2c	14 (51.8)	9 (33.3)	7 (25.9)	1 (3.7)
pT3a	3 (11.1)	3 (11.1)	3 (11.1)	1 (3.7)

Total	27 (100)	13 (48.1)	10 (37.0)	2 (7.4)

Gleason score				
2–6	19 (70.3)	3 (11.1)	2 (7.4)	0 (0)
7	7 (25.9)	9 (33.3)	7 (25.9)	1 (3.7)
8–10	1 (3.7)	1 (3.7)	1 (3.7)	1 (3.7)

Total	27 (100)	13 (48.1)	10 (37.0)	2 (7.4)

Lymph node classification				
N_0_	27 (100)
N_1_	0 (0)

**Table 2:** The pathological stages of bladder primary tumours.

Variable	Prostate Ca +		Prostate Ca −	
	Number of patients	Percentage of total	Number of patients	Percentage of total
T stage (TNM)				
pTa	1	3.7	4	7.4
pT1	2	7.4	5	9.2
pT2a	2	7.4	10	18.5
pT2b	4	14.8	2	3.7
pT3a	8	29.6	12	22.2
pT3b	1	3.7	5	9.2
pT4a	8	29.6	8	14.8
pT4b	1	3.7	0	0
pTis	0	0	8	14.8
Grade				
G1	0	0	2	3.7
G2	3	11.1	3	5.5
G3	24	88.9	49	90.7
Lymph node classification				
N_0_	24	88.9	47	87.0
N_1_	3	11.1	7	13.0
